# Oral Health and Quality of Life in Type 2 Diabetic Patients: Key Findings from a Romanian Study

**DOI:** 10.3390/jcm14020400

**Published:** 2025-01-10

**Authors:** Ramona Dumitrescu, Vanessa Bolchis, Simona Popescu, Adriana Ivanescu, Adrian Bolos, Daniela Jumanca, Atena Galuscan

**Affiliations:** 1 Translational and Experimental Clinical Research Centre in Oral Health, Department of Preventive, Community Dentistry and Oral Health, “Victor Babes” University of Medicine and Pharmacy, 300040 Timisoara, Romania; dumitrescu.ramona@umft.ro (R.D.); vanessa.bolchis@umft.ro (V.B.); jumanca.daniela@umft.ro (D.J.); galuscan.atena@umft.ro (A.G.); 2 Clinic of Preventive, Community Dentistry and Oral Health, Department I, “Victor Babes” University of Medicine and Pharmacy, Eftimie Murgu Sq. no 2, 300041 Timisoara, Romania; 3 Second Department of Internal Medicine, “Victor Babes” University of Medicine and Pharmacy, 300041 Timisoara, Romania; adriana.ivanescu@umft.ro; 4 Department of Diabetes, “Pius Brinzeu” Emergency Hospital, 300723 Timisoara, Romania; 5 Department of Oral Rehabilitation, Faculty of Dental Medicine, Specialization of Dental Technology, “Victor Babes” University of Medicine and Pharmacy, 300041 Timisoara, Romania; bolos.adrian@umft.ro

**Keywords:** diabetes mellitus, oral health, quality of life, knowledge, behaviors, OHIP-14

## Abstract

**Background:** Diabetes mellitus is associated with various oral health complications that can negatively impact quality of life. Despite evidence of the relationship between diabetes and oral health issues, limited research exists on the perceptions, behaviors, and oral health-related quality of life (OHRQoL) of diabetic patients in Romania. This study aims to evaluate self-reported oral health, knowledge, behaviors, and OHRQoL among diabetic patients in western Romania. **Methods:** A cross-sectional study was conducted in early 2024 at Pius Brinzeu County Emergency Hospital, Timisoara, involving 121 patients with type 2 diabetes. Data were collected using self-administered questionnaires that assessed oral health status, behaviors, and OHRQoL, with the OHIP-14 instrument employed for quality-of-life measurement. Statistical analyses included descriptive statistics, multiple linear regression, and correlation analyses to identify the predictors of oral health knowledge and OHRQoL. **Results:** Participants (mean age: 63.24 years; 52.1% female; 60.5% urban residents) reported significant oral health challenges. Over half (56.2%) did not visit a dentist regularly, with financial constraints identified as the primary barrier (23.5%). Oral hygiene practices were suboptimal: only 38.0% brushed twice daily, while 78.5% used fluoride toothpaste. Auxiliary hygiene tools, such as mouthwash and toothpicks, were rarely used (13.22% and 11.57%, respectively). Regression analyses identified the significant predictors of oral health knowledge, including tooth mobility (β = 0.33, *p* < 0.01) and brushing frequency (β = −0.18, *p* < 0.05). The mean OHIP-14 score (0.55) indicated a moderate impact on OHRQoL, with domains such as psychological discomfort and social disability revealing nuanced challenges. **Conclusions:** Diabetic patients in Romania face oral health challenges and care barriers, emphasizing the need for preventive strategies, oral health education, and integrated diabetes care. Addressing these gaps can improve oral health outcomes and overall quality of life in this vulnerable population.

## 1. Introduction

Systemic diseases can influence oral health both directly through pathological processes and indirectly through behavioral changes caused by the disease or its treatment. Likewise, changes in oral health can impact overall systemic well-being [1].

Extensive research underscores the global prevalence of diabetes, with 463 million individuals affected as of 2019, marking a notable surge over the past quarter-century [2,3]. Diabetes significantly impacts overall health, reducing life expectancy and increasing the risk of severe conditions such as cardiovascular disease, renal dysfunction, and malignancies. It is also strongly associated with compromised oral health, with type 2 diabetes patients facing a higher risk of oral complications, often linked to the degree and duration of hyperglycemia [4]. Observational studies link diabetes to conditions like periodontal disease, dental caries, oral infections, oral cancer, salivary dysfunction, and sensory disorders. Clinical research also suggests that periodontal health may affect glycemic control, while anti-diabetic medications can impact oral tissues [5]. Moreover, the interplay between oral health and chronic diseases such as diabetes has been extensively studied, with the findings indicating substantial evidence of mutual influence. For example, type 2 diabetes mellitus (T2DM) has been strongly associated with periodontal disease, both as a risk factor and as a condition exacerbated by poor glycemic control. These correlations underscore the critical need for integrating dental and medical care for patients with chronic diseases [6,7]. Although not frequently emphasized in diabetes care, even mildly elevated blood glucose levels can negatively impact oral health, leading to the development of various oral diseases and conditions [8], with periodontal disease being the most common [9]. However, the specific mechanisms connecting diabetes and periodontitis remain unclear due to a lack of experimental evidence from clinical studies [10]. Other prevalent issues include pronounced xerostomia, characterized by a reduction or absence of saliva, which can exacerbate dental caries or tooth decay. Additionally, individuals with diabetes are more susceptible to oral candidiasis, an infection caused by the yeast fungus *Candida albicans*, as well as lichen planus, a chronic inflammatory condition affecting the oral mucosa. Moreover, diabetic individuals may experience the distressing symptoms of burning mouth syndrome, a chronic oral pain disorder, further exacerbating their oral health challenges and potentially leading to tooth loss. These findings underscore the intricate interplay between systemic health factors like diabetes and oral health outcomes, necessitating comprehensive care approaches to mitigate associated risks and improve overall well-being. Emerging evidence also highlights the systemic pathways linking chronic inflammatory conditions like diabetes and periodontal disease, driven by shared biological mechanisms such as heightened inflammatory responses and dysregulated immune pathways. Reviews indicate that this connection extends beyond traditional oral health complications, suggesting that improving periodontal health can have a beneficial effect on systemic disease management, including glycemic control in diabetic patients [6,7].

To maximize the potential benefits of periodontal treatment, current guidelines [11] recommend that individuals with diabetes prioritize optimal oral hygiene practices and schedule regular dental check-ups to prevent periodontal disease and maintain overall oral health [12,13].

OHRQoL reflects the impact of oral health on physical, psychological, and social well-being, influencing activities like eating, speaking, and social interactions [14,15,16]. The OHIP-14, a short-form adaptation of the OHIP-49, is a practical and validated tool for measuring OHRQoL, maintaining excellent reliability [16].

Interest in quality of life (QoL) is increasing, with experts recognizing its multidimensional nature. In this context, it is crucial to assess the impact of oral health on QoL, especially among patients with chronic conditions that can influence oral health [17]. The quality of life (QoL) of patients with diabetes mellitus (DM) is lower than that of the general population, influenced by factors such as poor diabetes control, complications of the disease, fear of the unknown, concerns about family and social repercussions, and anxiety associated with undergoing diagnostic procedures and trying new treatment [18]. The interaction between diabetes and oral disorders makes the understanding of oral health-related quality of life (OHRQoL) in diabetic patients a crucial concern. OHRQOL is a relatively new but rapidly growing phenomenon that has emerged over the past two decades. According to the World Health Organization (WHO), quality of life is defined as “an individual’s perception of their position in life within the context of the culture and value system in which they live, and in relation to their goals, expectations, standards, and concerns”. Several studies have shown that oral disorders can negatively impact quality of life; however, their specific effect on diabetic patients remains under-researched [19]. As stated by the World Health Organization, a shift has been identified in the perception of health from the mere absence of disease and disability towards complete physical, mental, and social well-being [20]. This change took place in the second half of the 20th century, with the WHO playing an important role in defining the concept of health-related quality of life (HRQoL), which is defined as an individual’s subjective sense of well-being, encompassing overall happiness and life satisfaction, and later the concept of oral health-related quality of life (OHRQoL) [21]. This understanding has prompted a shift from a purely biomedical model to a biopsychosocial approach. The literature now includes a broader perspective based on Locker’s model, which examines oral health’s impact on quality of life [17,22].

Following the publication of the PREDATORR study [23] results, which assessed the prevalence of diabetes in Romania’s adult population and was completed in early 2014, it was revealed that the actual prevalence of diabetes in our country exceeds 11% among individuals aged 20 to 79 [24]. However, in Romania, there is a notable gap in understanding regarding the oral health knowledge, perceptions, and habits of those living with diabetes. To address this issue, the purpose of this study is to assess the oral health status, quality of life, knowledge, and behaviors of individuals with diabetes in Romania. By gaining insight into these aspects, we aim to contribute to more effective preventive strategies and improve the overall quality of care for people with diabetes. This study seeks to explore the perceived oral health status, common dental problems, levels of oral health knowledge, and behaviors, quality of life, as well as the factors influencing dental visits and barriers to accessing dental care for those living with diabetes in Romania.

## 2. Materials and Methods

### 2.1. Study Design and Sample Size

This observational, cross-sectional study was conducted at the Outpatient Diabetes Care Facility of the Pius Brinzeu County Emergency Hospital in Timisoara between February and March 2024. From a total of 160 diabetic patients attending scheduled appointments, 147 individuals (77 women, 70 men) were initially included, while 13 patients declined participation for personal reasons. Participants who reported having type 1 diabetes or were uncertain about their diabetes type were excluded from the sample, resulting in a final sample size of 121 participants. This decision was made to ensure a more focused and manageable sample size, as this study’s design aligns more closely with a pilot observational study. A smaller, more targeted sample was deemed appropriate for the objectives of this research. To estimate the sample size required for this study, we took into consideration the patient flow at the Outpatient Diabetes Care Facility of the Pius Brinzeu County Emergency Hospital in Timisoara. On average, each diabetologist at the center conducts around 2000 consultations annually, amounting to approximately 150 patient visits per month. This includes both initial and follow-up consultations, given the chronic nature of diabetes, which necessitates regular monitoring and prescription management. For the purpose of this study, one diabetologist (S.P.) was involved, ensuring continuity and representativeness in the sample of patients receiving diabetic care in this setting. These factors were integral to ensuring that the sample size accurately reflected the diabetic population served by the clinic and aligns with regional prevalence rates.

The sample size was calculated using G*Power software (3.1) based on a point biserial correlation model, aiming to detect significant associations between T2DM, periodontal disease, and quality of life domains measured by OHIP-14. Assumptions included a conservative estimate of 50% for the prevalence of oral health issues among diabetic patients, a 95% confidence level, and a 5% margin of error. To account for non-responses, a 20% adjustment was applied, resulting in a target sample size of 147 participants. Stratified random sampling, considering age, gender, and residential area (urban vs. rural), ensured representativeness. The final sample aligned with the regional urban-rural population distribution in western Romania. This sample calculation also accounted for a finite population from the western region of Romania, aligning with regional prevalence data.

This study was designed to investigate the relationship between diabetes, oral health, and the quality of life in diabetic patients. The target population includes diabetic individuals from the western region of Romania, where the prevalence of diabetes is 8.2% according to the PREDATORR study.

### 2.2. Inclusion/Exclusion Criteria

Individuals aged 18 and older with a confirmed diagnosis of type 2 diabetes were included in the study. Participants with comorbidities commonly associated with diabetes, such as hypertension, thyroid disorders, and ischemic cardiopathy, were included, as these conditions are prevalent among individuals with type 2 diabetes and can influence oral health. However, individuals with severe cognitive impairments or psychiatric conditions that could hinder their ability to provide informed consent were excluded. Additionally, patients requiring hospitalization during the study period or those residing in institutional settings were excluded to maintain the accuracy and reliability of the collected data.

### 2.3. Ethical Approval and Manuscript Preparation

All participants provided informed consent, with none involved in the study’s development. This research adhered to the principles of the Declaration of Helsinki (2013 version) and received approval from the Ethics Committee of the University of Medicine and Pharmacy Victor Babes, Timisoara, Romania (No. 05/30.01.2024).

Linguistic editing assistance for the development of this manuscript was provided using an artificial intelligence-based tool to improve the clarity and fluency of English expression. This tool was used solely for linguistic enhancement and did not modify or interpret the scientific content.

### 2.4. Data Collection

Two trained and experienced researchers (S.P. and R.D.) distributed an information sheet to potential participants, detailing the study’s purpose and addressing any questions they had. Participation was entirely voluntary, and individuals who met the inclusion criteria and agreed to join the study were given a consent form along with a self-administered questionnaire to complete while waiting for their medical appointment. Written consent was obtained from each participant, and it took approximately 8 to 15 min to fill out the questionnaire. The study questionnaire was designed based on a thorough review of the existing literature [10,25] and prior research [26,27]. It included 18 questions covering both individual factors, as well as health behaviors (personal health practices, interactions with medical care such as oral health information provided by healthcare professionals, and the use of healthcare services like dental visits). The development of the questionnaire was guided by Andersen’s behavioral model of health services utilization to evaluate the factors that affect access to dental services for individuals with diabetes [28]. The questions were divided into key areas, focusing on perceived oral health status, oral health-related quality of life (OHRQoL), knowledge of oral health, attitudes toward diabetes and oral health, oral care practices, barriers to accessing dental services, information related to diabetes management and oral health, as well as demographic and health-related characteristics of the participants. To assess knowledge of the connection between diabetes and oral health, the questionnaire included targeted questions about common oral health complications associated with diabetes. These questions evaluated the participants’ awareness of potential risks such as gingival bleeding, delayed wound healing, dry mouth (xerostomia), and the higher likelihood of periodontal disease due to elevated blood glucose levels. Responses were rated on a scale from ‘very well’ to ‘poor’ to gauge the participants’ depth of understanding. Additionally, questions about prior discussions with healthcare providers regarding oral health risks were included to determine if participants received specific education on this topic. This structured approach allowed us to measure both general awareness and specific knowledge related to diabetes and oral health.

Demographic factors, including five variables—gender, age, place of residence, duration of diabetes, and education level—were evaluated at the start of the study for each participant.

### 2.5. Oral Health Quality of Life

Oral Health-Related Quality of Life (OHQoL) was assessed using the Romanian version of the 14-item Oral Health Impact Profile (OHIP-14) [29]. The OHIP-14 encompasses seven domains, including Functional Limitation (e.g., difficulties with pronunciation and changes in taste), Physical Pain (e.g., oral pain and discomfort while eating), Psychological Discomfort (e.g., self-consciousness and tension due to oral health issues), Physical Disability (e.g., diet dissatisfaction and interrupted meals), Psychological Disability (e.g., challenges in relaxation and embarrassment), Social Disability (e.g., irritability and difficulties in routine activities), and Handicap (e.g., reduced life satisfaction and overall functionality due to oral health problems). Participants rated their experiences on a 4-point Likert scale as follows: very often = 4, often = 3, sometimes = 2, rarely = 1, and never = 0. The total score ranged from 0 to 56, with lower scores reflecting a higher OHRQoL.

The OHIP-14 has been validated as a reliable instrument for assessing oral health-related quality of life, with a strong internal consistency (α = 0.88). This short-form version captures over 90% of the variability measured by the original OHIP-49, ensuring practicality without compromising reliability [16].

### 2.6. Oral Hygiene Practices

Patients were asked questions regarding the type of toothpaste they used and their use of auxiliary oral hygiene tools. The type of toothpaste was coded as 0 (other types) or 1 (fluoride toothpaste), and the use of auxiliary hygiene tools was coded as 0 (no) or 1 (yes).

### 2.7. Statistical Analysis

The data were analyzed using the Statistical Package for the Social Sciences (SPSS) Version 23. Descriptive statistics, including mean and standard deviation for continuous variables and frequency counts with percentages for categorical variables, were used to describe demographic, socio-economic, and health-related characteristics, as well as self-reported oral health status, OHRQoL, oral health knowledge, and behaviors.

For OHRQoL, the OHIP-14 severity score was calculated by summing the coded responses (0 = never, 1 = rarely, 2 = sometimes, 3 = often, 4 = very often) across the 14 items, yielding a score range from 0 (indicating the best perceived oral health) to 56, with higher scores reflecting poorer OHRQoL. An impact on OHRQoL was considered when responses of “sometimes”, “often”, or “very often” were given [16]. Binary logistic regression was employed to explore the factors associated with adequate oral health knowledge (model 1) and dental visits within the past 12 months (model 2). These models were based on covariates identified in previous studies [10] and guided by the Anderson model [28].

In this study, a *p*-value threshold of *p* < 0.05 was employed as the primary criterion for statistical significance. However, given the constraints of the sample size and effect size related to some variables, values reaching *p* < 0.07 were considered marginally significant. This approach aligns with the intent to highlight trends that, while not meeting the conventional threshold, may indicate clinically relevant associations.

## 3. Results

### 3.1. Demographic Characteristics

A total of 147 patients were invited to take part in this survey, with 121 successfully completing it, yielding a response rate of 92%. The participants ranged in age from 28 to 85 years, with an average age of 63.24 years (SD = ±10.16). The most frequently reported age was 64 years. In terms of gender distribution, 52.07% of the participants were female (N = 63), while 47.93% were male (N = 58). A majority of participants (60.5%) resided in urban areas, while 36.1% lived in rural areas, with 3.4% (N = 5) not reporting their residential area.

Regarding educational attainment, the largest proportion of participants (48.75%) had completed 10 grades of school, followed by 23.97% who had finished high school, and 11.57% with a university degree. Most participants (23.1%) had been diagnosed within the past 1 to 5 years, followed by 18.2% who had been diagnosed for 6–10 years. A smaller proportion, 17.4%, had been diagnosed for less than one year. The number of participants gradually declined as the duration increased, with 16.5% diagnosed for 10–14 years, and 11.6% diagnosed for 15–19 years. Those with a diagnosis of 20–24 years accounted for 8.3%. Very few participants had been diagnosed for 25–29 years (1.7%) or more than 30 years (2.5%). The demographic characteristics of the individuals with diabetes are outlined in Table 1.

**Figure 1:**
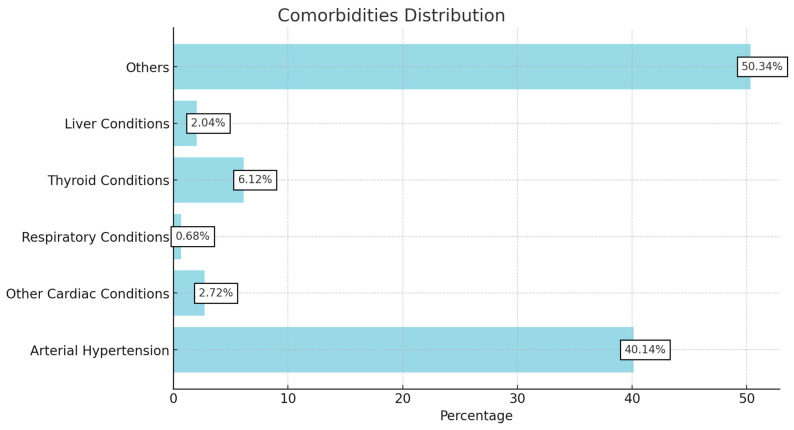
Comorbidities distribution in diabetic patients.

Among the 121 participants in the study, a notable proportion reported additional comorbidities commonly associated with diabetes. Specifically, 59 participants (40.1%) had arterial hypertension, which significantly increases cardiovascular risks when coupled with diabetes. Nine participants (6.1%) reported thyroid disorders, potentially complicating metabolic regulation and diabetes management. Additionally, four participants (2.7%) had ischemic cardiopathy, highlighting the heightened prevalence of cardiovascular diseases in the diabetic population. Other reported comorbidities were also respiratory conditions, glaucoma, and gut conditions (Figure 1).

### 3.2. Oral Health Status and Behaviors

Table 2 presents an overview of the participants’ self-reported oral health status, knowledge, and behaviors. Participants self-reported their oral health status, evaluated through a Likert scale focused on various oral health behaviors and perceptions.

Regarding oral health issues, 58.7% of participants reported no gum bleeding, and 64.5% did not experience sensitivity or pain in their teeth or gums. Dental mobility was reported by 33.9% of participants, while the majority, 57.0%, did not experience this issue.

### 3.3. Oral Health–Diabetes Relation

In terms of the relationship between diabetes and oral health, 53.7% of participants reported no dental or gum problems linked to their diabetes, and 71.1% indicated they did not experience increased gum bleeding after their diabetes diagnosis.

Results revealed that the majority (56.2%) did not visit a dentist regularly, with a mean score of 1.71 (SD = 1.04) on a scale where 1 indicated never visiting the dentist and 5 represented frequent visits (four or more times per year. Another 28.1% indicated that they visited the dentist once a year, while 9.1% attended dental checkups twice a year. A small percentage, 1.7%, reported visiting the dentist three times a year, and 5.0% stated they visited the dentist four or more times a year. These findings suggest that the majority of diabetic patients either neglect or infrequently seek dental care, which could have serious implications for their overall oral health management.

The average frequency of tooth brushing was reported as 2.46 times per day (SD = 0.96), with 38.0% of participants brushing their teeth twice a day. Another 28.1% brushed their teeth once a day, while 19.8% of the respondents brushed their teeth less than once per day, indicating a lower adherence to oral hygiene recommendations. A smaller group, 14.0%, reported brushing their teeth more than twice a day.

**Figure 2:**
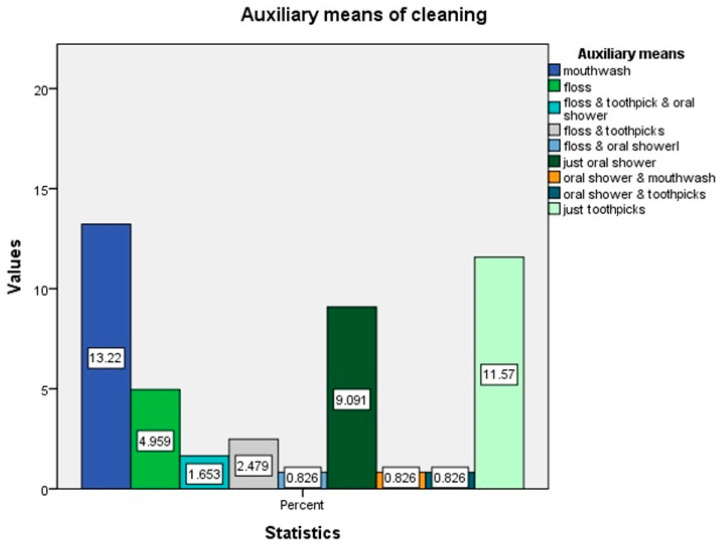
Descriptives for auxiliary tools used for oral cleaning.

Additionally, 78.5% of respondents used fluoride toothpaste (M = 1.21, SD = 0.41). Mouthwash was the most commonly used auxiliary oral hygiene method, with 13.22% of respondents indicating their use; toothpicks were used by 11.57% of individuals, making it the second most popular auxiliary tool, while 54.55% did not employ any auxiliary dental care methods beyond tooth brushing (Figure 2).

A multiple linear regression analysis was conducted to explore statistically significant associations with the dependent variable, participants’ self-reported understanding of the link between diabetes and oral health. The following factors were identified as statistically significantly associated with the multivariate model: dental mobility (β = 0.33, *p* < 0.01), frequency of tooth brushing (β = −0.18, *p* < 0.05), and whether participants had discussed oral health with their diabetologist (β = 0.13, *p* < 0.05). The regression model explained 17% of the variance in the participants’ knowledge regarding the connection between diabetes and oral health (F (8,112) = 3.03, *p* < 0.05, R^2^ = 0.178).

### 3.4. Oral Health Knowledge

The participants’ health knowledge was categorized as either strong or weak based on an evaluation of variables assessing their understanding. These variables included: understanding the link between diabetes and oral health issues, discussions with their doctor about the risks of oral health problems associated with diabetes, and recommendations received from their dentist in the context of a diabetes diagnosis. These items were rated using a Likert scale, and a global score was calculated as the mean of all items to assess overall oral health knowledge in the context of diabetes.

As shown in Table 3, the mean score for the participants’ oral health knowledge was 1.82 (SD = ±0.42), indicating a moderate level of understanding. Specifically, the average score for “Doctor’s debriefing about diabetes and oral health” was 1.67 (SD = ±0.63), reflecting variability in the frequency and quality of these discussions. The participants’ understanding of the link between diabetes and oral health had a mean score of 2.04 (SD = ±1.02), suggesting slightly higher awareness in this area. However, the mean score for “Special recommendations from the dentist” was 1.75 (SD = ±0.43), showing that recommendations were not consistently provided (Table 3).

### 3.5. Obstacles and Enablers for Accessing Dental Care

**Figure 3:**
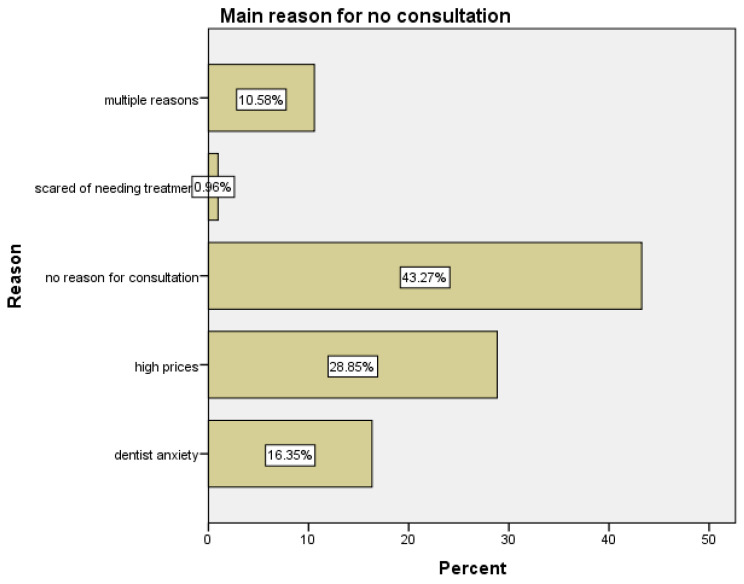
Reasons among participants for not visiting the dentist among participants.

Figure 3 outlines the reasons individuals choose not to consult a dentist, displaying the distribution of various factors influencing this decision. This item allowed participants to provide open-ended responses, which were subsequently categorized based on the frequency of certain themes or words. For example, responses mentioning the fear of visiting the dentist or stress associated with thinking about dentists were grouped under the variable “dentist anxiety”. Responses citing reasons related to the cost or the inability to afford treatment were classified under the variable “high prices”. Statements explaining general aversion to visiting the dentist, such as “don’t want to deal with the experience of going to the dentist,” were grouped under the variable “unpleasant experience at the dentist”. The decision not to include responses categorized as “scared of needing treatment” within the “dentist anxiety” category was deliberate, aiming to emphasize the distinction between anxiety triggered by the clinical environment or healthcare professionals (dentist anxiety) and anticipatory anxiety related to the treatment itself. The latter often involves economic considerations and the potential perception of harm, warranting its separate classification.

The most common reason, cited by 43.27% of respondents, was having no reason to consult, followed by high costs, which affected 28.85% of individuals. A significant portion (10.58%) provided other reasons for avoiding dental consultations, while dentist anxiety impacted 16.35%. Only a minimal number of participants indicated a fear of treatment as a main and singular reason for not consulting a dentist (0.96%). Some respondents also pointed to an unpleasant experience (2.47%) or a combination of high costs and fear/anxiety (1.63%) as deterrents. Smaller percentages indicated reasons like the high costs with no reason to consult (1.65%) or a mix of dentist anxiety and fear of treatment (0.82%). Additionally, concerns such as difficulty finding a dentist or fear of needing treatment were less frequently mentioned, each with percentages below 1%. All of the multiple reasons stated by the participants were taken into consideration as a global response under multiple reasons with a percent of 10.58% (Figure 3). These results emphasize that cost-related issues and a lack of perceived necessity are the predominant barriers to seeking dental care. These results highlight that a variety of personal, emotional, and financial factors influence individuals’ decisions to avoid dental consultations, with a notable proportion attributing their avoidance to reasons other than those typically explored in healthcare studies.

### 3.6. The Predictors of Having Adequate Oral Health Knowledge

This study identified several factors that had a statistically significant association with the participants’ understanding of the connection between diabetes and oral health issues. The dependent variable in the linear regression analysis was the participants’ self-reported oral health knowledge, as assessed through a structured questionnaire. Factors included gingival bleeding levels, tooth mobility, tooth sensitivity and pain, duration of diabetes diagnosis, OHIP-14 total score, and education level.

The analysis revealed that sensitivity or pain levels had a statistically significant association with self-reported oral health knowledge (β = 0.17, *p* < 0.05), accounting for 1.7% of the variability in the model. Similarly, mobility frequency showed a statistically significant association (β = 0.34, *p* < 0.01), contributing to 3.4% of the variance. The duration of diabetes diagnosis was negatively associated with oral health knowledge (β = −0.21, *p* < 0.05), accounting for 2.1% of the variability, indicating that individuals with a longer duration of diabetes were less likely to report higher levels of understanding. Additionally, tooth-brushing frequency had a statistically significant association with oral health knowledge (β = −0.16, *p* = 0.05), accounting for 1.6% of the variability, suggesting that higher tooth-brushing frequency was linked to better self-reported knowledge about the connection between diabetes and oral health. No statistically significant associations were observed for education level, gingival bleeding, or the use of additional hygiene tools.

To evaluate the direct perception of the link between oral health problems and diabetes, the dependent variable was the response to the item: “Have you ever experienced oral health problems due to a diabetes diagnosis?” This variable provided insight into the factors associated with self-reported oral health knowledge. A linear regression model demonstrated statistically significant associations with predictors such as gingival bleeding, tooth mobility, sensitivity or pain, the duration of diabetes diagnosis, and education level, collectively accounting for 4.5% of the total variability in the dependent variable (F(7,114) = 4.05, *p* < 0.001, R = 0.450). Among these, mobility frequency exhibited a statistically significant positive association (β = 0.34, *p* < 0.01), suggesting that participants with higher tooth mobility were more likely to report an understanding of the link between diabetes and oral health. Conversely, the duration of diabetes diagnosis was negatively associated with oral health knowledge (β = −0.21, *p* < 0.05), indicating that shorter disease duration correlated with higher reported understanding. Tooth-brushing frequency was similarly associated (β = −0.16, *p* = 0.05), reinforcing the relationship between regular oral hygiene practices and improved knowledge. The regression models did not find statistically significant associations for variables such as education level, gingival bleeding, or additional hygiene tool use in predicting oral health knowledge. These findings are summarized in Table 4.

### 3.7. Oral Health Quality of Life

The mean OHIP-14 score was calculated for the sample of 121 participants, with a total sum of responses amounting to 1132. Using the formula for the OHIP-14 score, the mean was found to be approximately 0.55, reflecting the average level of oral health-related quality of life in the study population.

In the Functional Limitation domain of the OHIP-14 questionnaire, participants were asked about speech and taste disturbances related to oral health. For the first item, concerning trouble pronouncing words due to oral health issues, the mean score was 0.60 (SD = 0.03), suggesting that pronunciation difficulties were uncommon. The data revealed a positively skewed distribution, with 53.7% of participants indicating they “Never” experienced this issue, while smaller groups reported “Very Rare” (5.8%) and “Occasional” (14.0%) difficulties. A small minority experienced more frequent issues (2.5%). The second item, addressing taste disturbances, showed a slightly higher mean score of 0.73 (SD = 1.01), with 43.8% of respondents reporting no taste issues and 16.5% reporting occasional impairment.

In the Physical Pain domain, responses to items about aching and discomfort while eating reflected that 47.9% of respondents “Never” experienced mouth pain, although 14.9% occasionally did. Similarly, 40.5% never felt discomfort while eating, but 18.2% experienced it occasionally. These findings indicate that, while pain and eating discomfort are infrequent, they do affect a considerable minority, potentially impacting their quality of life.

The Psychological Discomfort domain highlighted that 34.7% of participants never felt self-conscious due to oral health, while 17.4% reported occasional discomfort. Regarding distress linked to oral appearance, 42.1% of respondents never experienced it, but 17.4% noted occasional distress. These results show that psychological discomfort, while not widespread, affected some individuals and may influence social interactions.

In the Physical Disability domain, items focused on difficulties performing daily tasks and physical discomfort linked to oral health. Here, 47.9% reported no task-related issues, although 14.0% experienced occasional limitations. A similar pattern was observed for physical discomfort during eating or speaking, with 47.1% unaffected and 14.0% occasionally affected.

For Psychological Disability, items addressed feelings of frustration or helplessness. Item 9 revealed that 49.6% never felt psychologically hindered by oral health, while 14.9% felt so occasionally. Similar findings were noted for a related item, where 52.1% reported no psychological disability, and 13.2% reported occasional experiences.

The Social Disability domain, examining social restrictions due to oral health, showed that 45.5% reported no social difficulties, while 15.7% experienced them occasionally. Another item in this domain revealed similar results, with 50.4% unaffected and 13.2% occasionally affected.

Lastly, in the Handicap domain, which explored overall feelings of disadvantage, 47.9% reported never feeling handicapped, with 16.5% indicating occasional experiences. Item 14 showed similar results, with 42.1% unaffected and 14.9% occasionally affected (Table 5).

To explore the relationship between demographic and health variables, knowledge levels, and the participants’ OHIP-14 scores, we conducted a comprehensive analysis of these factors. The results suggest that higher education levels are positively associated with oral health knowledge and improved OHIP-14 scores, indicating that education may influence both knowledge and quality of life. Additionally, participants with type 2 diabetes, particularly those with a longer duration since diagnosis, reported lower OHIP-14 scores, suggesting that prolonged exposure to diabetes management may foster adaptation and reduce its impact on quality of life. Furthermore, oral health behaviors, such as regular dental visits and frequent brushing, were associated with higher knowledge levels and better OHIP-14 scores, underscoring the role of proactive oral health behaviors in enhancing quality of life among diabetic patients. These findings provide a broader understanding of how oral health knowledge and quality of life are interconnected across various participant characteristics.

The parametric Pearson correlations revealed several significant relationships concerning age, education, environment, oral health status, and specific oral health impacts among diabetic patients, as well as specific domains from OHIP-14 (Table 6).

The Pearson correlation analysis between the OHIP-14 domains and the variables of interest highlighted statistically significant relationships, represented by varying color intensities corresponding to the strength of the correlation. Strong positive correlations (e.g., r > 0.5) are marked with more intense warm colors (e.g., orange or red). For instance, significant relationships were observed between “Physical Disability” and “Psychological Disability” (r = 0.836, *p* < 0.01) and between “Social Disability” and “Handicap Domain” (r = 0.840, *p* < 0.01), emphasizing the interdependence between physical and psychosocial domains of oral health. Significant negative correlations, represented by cooler tones, included “Education” and “Functional Limitation” (r = −0.263, *p* < 0.05) and “Education” and “Physical Pain” (r = −0.213, *p* < 0.05), suggesting that higher levels of education were associated with the reduced impacts of oral health issues, potentially reflecting better awareness and access to care. Moderate correlations, such as “Bleeding” and “Sensitivity or Pain” (r = 0.429, *p* < 0.01), are represented by neutral tones (e.g., yellow or light orange), highlighting connections between physical symptoms and quality of life.

### 3.8. The Education Variable in Relation to the Domains and Other Social Factors

A significant negative correlation was found between education level and the duration since diabetes diagnosis (R = −0.23, *p* < 0.05), indicating that individuals with higher education levels had been diagnosed with diabetes for a shorter period of time, though the effect size was small r^2^ = 0.05. Education was also negatively correlated with the physical disability domain (r (93) = −0.21, *p* < 0.05), meaning as the level of education increased, the mean of physical disabilities caused by oral health lessened, with a small effect size of r^2^ = 0.04. Lastly, education was significantly and negatively correlated with the functional limitation domain (r (93) = −0.26, *p* < 0.05), indicating that as the education level increased, the level of functional limitations decreased, with a small effect size of r^2^ = 0.06.

### 3.9. Demographic, Oral Health Knowledge, and Oral Health Status in Relation to OHIP-14 Domains

There was also a negative correlation between psychological discomfort and sensitivity or pain reported by participants (r (93) = −0.27, *p* < 0.05), indicating that psychological distress increased when pain or sensitivity of gums occurred, and due to the nature of coding these variables, with a small effect size of r^2^ = 0.07. The same effect was observed between the sensitivity or pain reported and the physical disability domain (r (93) = −0.20, *p* < 0.05), with a small effect size of r^2^ = 0.04. Frequency of sensitivity or pain reported by the participants was also correlated with oral health knowledge (r (93) = 0.24, *p* < 0.05), indicating that as the overall oral health knowledge increased, the frequency of pain or sensitivity reported lessened, with a small effect of r^2^ = 0.05

Regarding bleeding frequency, significant correlations were found with the handicap domain (r (93) = −0.21, *p* < 0.05), meaning that as the frequency of bleeding increased, so did the mean level of handicap domain, with a small effect of r^2^ = 0.04. Another significant correlation regarding the frequency of bleeding was observed with the mean score of oral health knowledge (r (93) = 0.20, *p* < 0.05), meaning as oral health knowledge increased, the bleeding frequency decreased, with a small effect of r^2^ = 0.04.

No significant correlations were identified between the OHIP-14 domains and the variables of diagnostic duration, tooth brushing frequency, or tooth mobility in this sample.

To further examine the impact of oral health knowledge on quality of life, we conducted an additional analysis, grouping participants based on their self-reported knowledge of the diabetes–oral health link. Two groups were created: a high knowledge group, consisting of participants who rated their understanding as “Good” or “Very Good”, and a low knowledge group, consisting of those who rated their knowledge as “Satisfactory” or “Poor”.

The mean OHIP-14 score for the high knowledge group was 6.67, while the low knowledge group’s mean score was 9.34. Although a trend was observed where greater knowledge correlated with better oral health-related quality of life (indicated by lower OHIP-14 scores), a t-test comparison yielded a *p*-value of 0.20, suggesting that this difference was not statistically significant. This preliminary finding indicates a potential association between the increased awareness of oral health risks and improved quality of life, though further research with a larger sample size may be needed to confirm this relationship.

## 4. Discussion

To the best of our knowledge, this is the first study to assess the self-reported oral health status, knowledge, behaviors, and oral health quality of life of people living with diabetes in Romania. Participants were recruited from the western region of the country, with residences in both urban and rural areas. The most striking finding was that over half of the participants reported experiencing at least one oral health issue, yet significant gaps in knowledge about the relationship between diabetes and oral health were evident. Only 38.0% of participants reported brushing their teeth twice daily, and a minority used auxiliary hygiene tools like mouthwash (13.22%) or toothpicks (11.57%). These behaviors reflect suboptimal adherence to recommended oral hygiene practices. Despite the relatively low mean OHIP-14 score (0.55), indicating a smaller overall impact on OHRQoL, specific domains such as psychological discomfort and social disability highlighted nuanced challenges faced by a subset of participants. These findings emphasize the critical need for integrating oral health education into diabetes management programs to improve awareness, promote proactive behaviors, and enhance overall well-being. The sample was generally representative of the population data reported in the PREDATORR study, which provided the most recent epidemiological insights into diabetes in Romania. Specifically, the mean (SD) age of participants in our study was comparable to the national data, with 63.24 years (SD = ±10.16), years in our sample versus 59.7 (±13.9) years in the PREDATORR study. The median duration of diabetes in our study was 12.5 years, close to the national average of 13.1 years reported in PREDATORR.

Based on evidence from systematic reviews, a bidirectional relationship between diabetes and periodontal disease has been firmly established. As indicated by Seitz et al. [6], individuals with diabetes are at significantly higher risk for periodontal disease due to systemic inflammation and impaired immune response. Conversely, managing periodontal disease has been shown to improve glycemic control, supporting a synergistic approach to oral and systemic health management.

The prevalence of oral health issues may be linked to the presence of diabetes complications and the lower socio-economic status of the participants. In comparison to Romania, Australia (24%) [30] and Canada (21%) [31] report lower proportions of individuals rating their oral health as poor to fair, though it is important to note that the healthcare systems in these countries are different from Romania’s. This elevated percentage in Romania is understandable given that diabetes is known to predispose individuals to various oral health issues, as also highlighted in other international studies.

One potential explanation for the variation observed in Romania could be the presence or absence of chronic or acute diabetes-related complications among participants, as these have been associated with a higher likelihood of reporting poor oral health. Furthermore, over half of the participants in this study reported experiencing one or more oral health issues. This finding aligns with studies conducted in the Netherlands [32], where significant proportions of individuals with diabetes reported oral health problems, particularly periodontal issues, and other complications. In comparison, studies from Canada using data from the Canadian Community Health Survey (2003 and 2007–2008) reported slightly lower proportions of self-reported poor oral health among diabetics, highlighting potential differences in healthcare access, oral health awareness, or socioeconomic factors across populations. In our study, most of the participants reported having other chronic illnesses in addition to diabetes, like hypertension, ischemic cardiopathy, and thyroid disorders [31].

While studies have assessed quality of life in patients with diabetes, few have specifically examined OHRQoL in this group. The literature shows some uncertainty regarding the impact of diabetes on OHRQoL. While some studies suggest that diabetes negatively affects OHRQoL, particularly among older adults, others have found that physical and environmental aspects score lower than social and psychological domains. Consequently, psychological support and lifestyle changes are recommended for diabetic patients. However, several studies argue that diabetes itself does not directly affect OHRQoL; instead, it complicates oral health. Several studies have investigated the relationship between diabetes and oral health-related quality of life (OHRQoL). Nikbin et al. [33] found that the severity of oral health issues negatively impacts OHRQoL in diabetic patients, as measured by validated instruments like OHIP-14 and GOHAI. Similarly, Aschalew et al. [34] highlighted factors such as glycemic control and comorbidities as significant determinants of health-related quality of life among diabetic patients. Gomes et al. [35] further identified clinical predictors of poor OHRQoL in older adults with diabetes, emphasizing the role of oral health conditions and systemic complications [33,34,35]. However, the mean OHIP-14 severity score in our study (0.55) was substantially lower than that found in the Dutch study (2.5 ± 5.2), reflecting a smaller impact on OHRQoL in Romania. In a study conducted in the United States, the impact on OHRQoL was 47.7%. Other studies have reported prevalence rates, ranging from 22.5% to 34.4% in type 2 diabetes patients. These discrepancies may be attributed to cultural differences within the studied population, as well as to the methods used in the study [36]. Allen et al. demonstrated that diabetes had no significant impact on OHRQL in the group they studied, a finding consistent with the results of Sandberg et al.’s case-control study. Additionally, there is limited evidence for the awareness of the increased risk of periodontal disease among diabetic patients. Allen et al. found that only 33% of diabetic patients were aware of this elevated risk [37], while Sandberg et al. reported that just 17% of their study population had such awareness [38].

Other previous analysis from the literature also emphasized that patients who were better informed or had a solid understanding of the connection between diabetes and oral health were more inclined to adopt beneficial oral health behaviors [10]. However, in our study, only 75.2% of participants reported receiving oral health information from healthcare providers, a trend consistent with findings from other international studies [10]. This highlights a critical need for oral health education to be integrated into routine diabetes care. In Romania, this may be attributed to the fact that incorporating oral health education into diabetes care settings is still relatively new and poses challenges due to time limitations and the lack of specialized training or confidence among healthcare professionals in promoting oral health. As many healthcare providers are not adequately trained in oral health, this results in missed opportunities for patient education. Therefore, targeted training programs and clinical initiatives to enhance healthcare providers’ capacity to address oral health in diabetes management are necessary. Studies have shown that when patients receive oral health information from their care providers, they are more likely to engage in positive oral health behaviors, including routine dental check-ups [26,27]. A majority of the studies indicate that patients with diabetes often have low awareness about the link between diabetes and oral health. For example, in the study by Sadeghi et al. from Iran, only 36.5% were aware of the connection between oral health and diabetes, and most of the information came from dentists (65%) [19].

Similarly, Al Habashneh et al. [39] in Jordan found that 47.7% of people with diabetes recognized the link between periodontal disease and diabetes, with their primary source of information being healthcare providers like diabetes nurses and physicians [10]. This underscores the need for similar efforts in Romania to improve oral health outcomes for diabetic patients. Given the bidirectional relationship between diabetes and oral health, in particular periodontal disease, collaboration between oral health care professionals and diabetes care providers is crucial.

When examining oral health behaviors, 38.0% of participants reported brushing their teeth twice daily, while 28.1% brushed once a day, and 19.8% brushed less than once daily, reflecting suboptimal adherence to oral hygiene recommendations. A smaller proportion (14.0%) exceeded the recommended frequency by brushing more than twice daily. The majority (78.5%) reported using fluoride toothpaste, suggesting widespread adoption of this key oral health practice. However, 21.5% did not use fluoride toothpaste, potentially increasing their risk of dental issues, particularly given their vulnerability to diabetes-related oral health complications. Interdental cleaning appeared to be a low priority, with only 13.22% using mouthwash and 11.57% using toothpicks, while floss use was notably low and not explicitly reported, highlighting the need for increased emphasis on auxiliary oral hygiene practices in this population. Research has shown that the lack of interdental cleaning is linked to poorer blood glucose control and various oral health issues [40,41]. Additionally, there is evidence that interdental cleaning, alongside regular brushing, can help reduce gingivitis, plaque, or both [42]. There is considerable variation across different countries regarding brushing habits and the frequency of dental visits. For instance, the study by Bangash et al. (2011) [43] in Pakistan reported high brushing rates (86%), but dental visits were low, reflecting a significant gap between hygiene practices and professional care. In contrast, Lee et al. (2009) [44] in South Korea reported that 90.6% of participants brushed their teeth regularly, and 45.3% had dental visits in the past six months, indicating better adherence to both hygiene and professional care compared to other regions [10]. Therefore, it is evident that improved communication about the importance of regular flossing is essential for this at-risk population. These findings underscore the importance of promoting the use of fluoride toothpaste as part of regular oral hygiene routines to help prevent tooth decay and maintain better overall oral health in diabetic individuals. These findings suggest that while a majority of patients maintain relatively good oral hygiene, a notable proportion may not brush their teeth frequently enough, which could negatively impact their oral health, especially in a population at higher risk for complications due to diabetes.

In Romania, the rate of dental visits in the past 12 months was also reported to be lower than the findings from other countries, such as the UK (85.2%) [45], Sweden (85.1%) [38], the Netherlands (76%) [46], Australia (62.7%) [30], and the USA (72.7% and 65.8%) [47]. Studies from other countries have shown that dental attendance rates are generally lower among patients with diabetes compared to those without the condition. This is particularly concerning, as diabetes increases the risk of periodontal disease, which in turn negatively affects blood sugar control and exacerbates diabetes-related complications. The reduced frequency of dental visits among this group could be attributed to the prioritization of other health concerns, such as depression, mental health issues, and diabetes-related stress, which are often prevalent among diabetic patients.

Similar to previous research [48], a significant factor influencing dental visits in the past 12 months was a higher level of education. This may be due to the fact that individuals with more education tend to have a better understanding of oral health issues and treatments, which in turn leads to greater adherence to recommended oral health practices.

The findings from the present study further emphasize that financial barriers play a crucial role in limiting access to dental care. A significant portion of participants reported not visiting a dentist regularly, with 14.05% identifying high costs as the primary deterrent. This observation aligns with global trends, where economic constraints are a predominant obstacle to seeking dental care, particularly among low-income populations [49].

Scientific research in the medical field consistently highlights a key fact: health begins in the mouth. Today, maintaining good oral health is not only about the well-being of teeth but, as extensively demonstrated in the literature, serves as a foundation for overall bodily health and well-being [15]. Evidence from the literature shows that oral health can have wide-ranging systemic implications, potentially affecting multiple organs. For example, periodontal disease has been linked to insulin resistance and even more complex complications involving the cardiovascular system and neurodegenerative conditions. Therefore, improving oral health could have significant benefits for the body, aiding in the prevention of various diseases, improving quality of life, and having a positive impact on both individuals and society as a whole.

Age has also been identified as a factor influencing the health-related quality of life (HRQOL) in diabetic patients. While Hanninen et al. [50] concluded that age does not impact the HRQOL of diabetic individuals, another study found that patients under 40 years old experience significantly better quality of life compared to other age groups [50]. Moreover, it is recognized that men and women with diabetes encounter distinct challenges in managing their condition [51]. The findings from this OHIP-14 analysis in our study underscore that while most respondents report minimal impact from oral health issues across functional, physical, psychological, and social domains, a significant minority do experience occasional or recurrent challenges. These experiences, particularly in domains like psychological discomfort and social disability, highlight the nuanced impact of oral health on quality of life beyond physical symptoms. The occasional limitations reported by a subset of respondents suggest the need for targeted interventions, especially for those facing recurring issues. Such interventions could include counseling on coping mechanisms and strategies to reduce self-consciousness related to oral health appearance, potentially enhancing social and psychological well-being for affected individuals. An additional analysis was conducted to examine the relationship between patients’ awareness of the link between diabetes and oral health and their OHIP-14 scores. Our findings suggest that individuals with higher levels of knowledge on this connection report lower OHIP-14 scores, indicating a potentially improved oral health-related quality of life. This suggests that greater awareness of the impact of diabetes on oral health may correlate with more proactive behaviors, thereby reducing the negative impacts of oral health issues on daily functioning and well-being. Our findings regarding the impact of diabetes awareness on oral health-related quality of life are supported by recent studies that utilized the OHIP-14 to measure similar outcomes in diabetic populations. A study by Oluwatoyin et al. (2024) [52] examined self-reported oral health and OHIP-14 scores among diabetic patients, revealing that patients with a higher awareness of oral health risks had significantly better oral health-related quality of life. This relationship was further substantiated through clinical examinations, which assessed oral hygiene status and mucosal lesions, indicating a tangible benefit of oral health education on patient-reported outcomes. Furthermore, research by Poudel et al. (2020) [27] highlights similar findings in Australia, where diabetic patients with a greater awareness of the connection between diabetes and oral health demonstrated better self-reported oral health and improved OHIP-14 scores. The study emphasizes that increased oral health education among diabetic patients is associated with fewer perceived impacts on daily life and enhanced overall well-being. Future studies could expand on these findings by exploring interventions focused on enhancing patient education to assess the potential for quality-of-life improvements.

The concept of empowerment [53], which the WHO describes as a process where individuals gain control over decisions affecting their health, has recently gained attention, including among diabetic patients. This approach is believed to enhance the patient–doctor relationship and encourage patients to follow appropriate medical advice more closely. However, to foster an empowered diabetic population, comprehensive patient education is essential [54]. Beyond managing risk factors, which has been extensively studied, patients should be better informed about potential oral health issues associated with diabetes, such as gum disease, oral infections, dry mouth, and delayed healing of mouth sores. Poor treatment adherence is a common challenge among diabetic patients. Therefore, increasing patient education about the link between diabetes and oral health could help overcome this barrier, ultimately improving their overall well-being in the long term. Oral diseases, including periodontal disease, are closely linked with non-communicable diseases such as diabetes and cardiovascular diseases. Evidence from the umbrella review by Botelho et al. [7] highlights the WHO’s call for integrating oral health into universal healthcare policies, emphasizing prevention and interdisciplinary care. This integration is especially vital in addressing shared risk factors like smoking, poor diet, and socioeconomic inequities, which exacerbate both oral and systemic health conditions.

This study stands out for its methodology and valuable contributions to the field. The use of the validated OHIP-14 instrument provided a reliable and comprehensive assessment of oral health-related quality of life, ensuring the accuracy of the findings. Moreover, the inclusion of a representative population through stratified random sampling captures the diversity of individuals with type 2 diabetes in an under-researched region. By addressing a significant gap in the literature, this research offers critical insights into the oral health challenges faced by this population, serving as a foundation for public health initiatives and future studies.

Despite these strengths, this study has several limitations that should be considered when interpreting the findings. First, the study’s cross-sectional design limits the ability to establish causality between diabetes and oral health outcomes. The reliance on self-reported data may also introduce reporting bias, as participants may have either overestimated or underestimated their oral health status and behaviors. While this study relied on self-reported oral health data to ensure participant convenience, future studies would benefit from incorporating objective oral health assessments to provide a more comprehensive understanding of oral health status in diabetic populations. These assessments could include standardized periodontal examinations, such as measuring probing pocket depths (PPD), clinical attachment loss (CAL), and bleeding on probing (BOP), which would allow for a detailed evaluation of periodontal health—a key concern in diabetic populations. Additionally, dental caries assessments through the DMFT index (Decayed, Missing, and Filled Teeth) and salivary flow rate measurements could provide further insights into caries risk and xerostomia prevalence among diabetic patients. The incorporation of these objective measures would not only enhance the reliability of the findings but also provide a comprehensive picture of the oral health status in this vulnerable population.

Additionally, the sample was drawn from a single region in Romania, which may limit the generalizability of the findings to other geographic areas or populations. Although this sample provides preliminary insights, the findings should be interpreted with caution due to the limited size, positioning this study as a foundation for further research with larger cohorts. Another limitation of this study is the absence of a control group, which would have allowed for direct comparisons between diabetic and non-diabetic populations. As this was a cross-sectional, observational study, our focus was on evaluating oral health-related outcomes within the diabetic patient group. The absence of a control group is a recognized limitation in observational studies [55,56], as it can affect the ability to draw causal inferences. Future research can incorporate a control group of non-diabetic individuals to more rigorously assess the specific impact of diabetes on oral health and related quality of life. Such a design would enable a more comprehensive understanding of the differences in oral health outcomes attributable to diabetes. While this study highlighted a lack of oral health information provided by healthcare providers, it did not explore the reasons behind this gap. Although financial barriers were identified as a major obstacle to dental care, this study did not explore the specific economic challenges faced by patients in detail. A more comprehensive analysis of socio-economic factors could offer deeper insights into the affordability of oral health services for diabetic patients.

Finally, the study’s relatively small sample size and the exclusion of individuals with severe cognitive or psychiatric conditions may have further impacted the results. Future research should aim to address these limitations by utilizing longitudinal designs, larger and more diverse samples, and objective clinical assessments of oral health.

## 5. Conclusions

This study highlights the significant oral health challenges faced by diabetic patients in Romania, including poor self-reported oral health, low adherence to recommended oral hygiene practices, and infrequent dental visits, often exacerbated by financial and educational barriers. The findings underscore the critical need to integrate oral health education into routine diabetes care, emphasizing the bidirectional relationship between oral and systemic health. Research further highlights that enhancing oral health knowledge is crucial for improving self-care practices related to oral hygiene. Despite well-documented connections between diabetes, periodontal disease, and existing oral care recommendations, individuals with diabetes often lack sufficient knowledge, awareness, and adherence to proper oral health practices. Although the overall impact on quality of life was modest, psychological and social challenges were notable for some participants, highlighting the importance of holistic, patient-centered care. Addressing these gaps requires targeted preventive strategies, such as regular oral health evaluations, enhanced education for both patients and healthcare providers, and improved access to affordable dental care. By adopting a multidisciplinary approach, public health initiatives can significantly improve the oral health and overall well-being of diabetic individuals in Romania.

## Figures and Tables

**Table 1:** Demographic characteristics (N = 121).

Variable		N (%)
Gender	Male	58 (47.93)
	Female	63 (52.07)
Age	Mean (SD) Range	63.24 (±10.16) 28–85
Place of residence	Urban Rural	73(60.5) 43 (36.1)
Type of education	8th Grade 10 Grade Highschool Vocational School University Degree	18 (14.88) 59 (48.75) 29 (23.97) 1 (0.83) 14 (11.57)
Frequency of Dental Visits	Never Once a year Twice a year Three times a year Four or more times a year	68 (56.20) 34 (28.10) 11 (9.09) 2 (1.65) 6 (4.96)

**Table 2:** Self-reported oral health status, knowledge, and behaviors.

Variable		N
Bleeding (yes) Sensitivity OR Pain (yes) Mobility (yes) Dental problems because of Diabetes (yes)		50 43 41 37
Knowledge regarding link between Diabetes and oral health	Adequate Inadequate	44 77
Dental visit in the last 12 months	Regular visit No visit	53 68
Brushing frequency/day	Twice a day or more Once a day Less than once a day	63 34 24

**Table 3:** Descriptives for oral health knowledge.

Variable	Mean	Std Deviation	Variance
Oral health knowledge Doctor’s debriefing about diabetes & oral health	1.82 1.67	±0.42 ±0.63	0.18 0.40
Understanding the link between diabetes & oral health Special recommendations from dentist	2.04 1.75	±1.02 ±0.43	1.05 0.18

**Table 4:** Variables statistically associated in a multivariate model for oral health knowledge as the dependent variable.

	Statistical Associations	
Variable	Beta	T
Bleeding Sensitivity or Pain Mobility Teeth Washing Frequency	−0.11 0.12 0.34 ** −0.16	−1.25 1.26 3.81 −1.8
Other Hygiene Means Education	0.00 −0.03	0.02 −0.36
Duration of diagnosis	−0.21 **	−2.44

Note: Dependent variable: Oral health knowledge as assessed through the questionnaire; ** *p* < 0.01.

**Table 5:** OHIP-14-descriptive statistics by domain and item.

Domain	Mean.		Std. Deviation	Variance
Functional Limitation	Item 1 Item 2	0.60 0.73	1.03 1.01	1.06 1.02
Physical Pain	Item 3 Item 4	0.70 0.89	1.3 1.10	1.06 1.2
Psychological Discomfort	Item 5 Item 6	1.22 0.85	1.38 1.0	1.91 1.17
Physical Disability	Item 7 Item 8	0.75 0.75	1.10 1.10	1.21 1.21
Psychological Disability	Item 9 Item 10	0.62 0.61	0.94 0.98	0.88 0.97
Social Disability	Item 11 Item 12	0.77 0.60	1.08 0.92	0.17 0.85
Handicap domain	Item 13 Item 14	0.72 0.82	1.04 1.06	1.09 1.12

**Table 6:** Pearson correlations between demographic factors, environment, oral health status, and OHIP-14 domains among diabetic patients.

	Pearson Correlations Between Demographic Factors, Environment, Oral Health Status, and OHIP-14 Domains Among Diabetic Patients														
Variables	1	2	3	4	5	6	7	8	9	10	11	12	13	14	15
Oral_health_knowledge	1	−0.022	−0.046	−0.039	−0.039	−0.031	0.012	−0.022	0.078	−0.131	0.149	0.2	0.243	0.159	−0.016
Functional_Limitation	−0.022	1	0.623	0.469	0.661	0.761	0.627	0.515	−0.263	−0.079	−0.167	−0.041	−0.089	−0.087	0.329
Physical_Pain	−0.046	0.623	1	0.557	0.643	0.721	0.68	0.606	−0.213	0.041	−0.043	−0.064	−0.125	0.086	0.169
Psychological_Discomfort	−0.039	0.469	0.557	1	0.691	0.659	0.594	0.668	−0.06	0.09	−0.106	−0.152	−0.278	0.011	0.121
Physical_Disability	−0.039	0.661	0.643	0.691	1	0.836	0.717	0.759	−0.175	0.075	−0.107	−0.16	−0.207	−0.068	0.207
Psychological_Disability	−0.031	0.761	0.721	0.659	0.836	1	0.78	0.695	−0.127	−0.013	−0.147	−0.012	−0.189	−0.038	0.25
Social_Disability	0.012	0.627	0.68	0.594	0.717	0.78	1	0.84	−0.17	0.64	−0.107	−0.144	−0.198	−0.04	0.155
Handicap_Domain	−0.022	0.515	0.606	0.688	0.759	0.695	0.84	1	−0.138	0.061	−0.151	−0.215	−0.194	−0.006	0.156
Education	0.078	−0.263	−0.213	−0.06	−0.175	−0.127	−0.17	−0.138	1	−0.238	0.042	−0.049	−0.007	0.02	−0.14
For_how_long_d_diagnostic	−0.131	−0.079	0.041	0.09	0.075	−0.013	0.064	0.061	−0.238	1	0.046	−0.166	−0.08	−0.056	0.015
TeethWashingFreq	0.149	−0.167	−0.043	−0.106	−0.107	−0.147	−0.107	−0.151	0.42	0.46	1	0.125	0.124	0.211	−0.126
Bleeding	0.2	−0.041	−0.064	−0.152	−0.16	−0.012	−0.144	−0.215	−0.049	−0.166	0.125	1	0.429	0.183	0.194
SensitivityOrPain	0.243	−0.089	−0.125	−0.278	−0.207	−0.189	−0.198	−0.194	−0.007	−0.08	0.124	0.429	1	0.294	−0.032
Mobility	0.159	−0.087	0.086	0.011	−0.068	−0.038	−0.04	−0.006	0.02	−0.056	0.211	0.183	0.294	1	−0.052
Flour	−0.016	0.329	0.169	0.121	0.207	0.25	0.155	0.156	−0.14	0.15	−0.126	0.194	−0.032	−0.052	1

## Data Availability

The data presented in this study are available on request from the corresponding author.
